# Infertility treatments and risk of breast benign diseases: a case‒control study

**DOI:** 10.1186/s12905-024-03429-w

**Published:** 2024-11-01

**Authors:** Ashraf Moini, Sadaf Alipour, Zahra Zandi, Arezoo Maleki-Hajiagha, Ladan kashani, Fatemeh Shakki katouli, Maryam Farid Mojtahedi, Leila Bayani, Mahboubeh Abedi

**Affiliations:** 1 Endocrinology and Female Infertility Unit, Arash Women’s Hospital, Tehran University of Medical Sciences, Tehran, Iran; 2 Department of Endocrinology and Female Infertility, Reproductive Biomedicine Research Center, Royan Institute for Reproductive Biomedicine, ACECR, Tehran, Tehran Iran; 3 Breast Disease Research Center (BDRC), Cancer Institute, Tehran University of Medical Sciences, Tehran, Iran; 4 Department of Surgery, Arash Women’s Hospital, Tehran University of Medical Sciences, Tehran, Iran; 5 Department of Obstetrics and Gynecology, Arash Women’s Hospital, Tehran University of Medical Sciences, Tehran, Iran; 6 Department of Anatomy, School of Medicine, Tehran University of Medical Sciences, Tehran, Iran; 7 Students’ Scientific Research Center (SSRC), Tehran University of Medical Sciences, Tehran, Iran; 8 Advanced Diagnostic and Interventional Radiology Research Center (ADIR), Tehran University of Medical Sciences, Tehran, Iran; 9 Department of Radiology, Arash Women Hospital, Tehran University of Medical Sciences, Tehran, Iran; 10 Department of Radiology, Ballarat Base Hospital, Ballarat, VIC Australia; 11 Arash Women’s Hospital, Rashid Ave, Resalat Highway, Tehranpars, Tehran Iran

**Keywords:** Infertility treatment, Ovarian-stimulating hormones, Assisted reproductive techniques, Ovulation induction, Breast benign diseases

## Abstract

**Background:**

Theoretically, endocrine fluctuations occurring during infertility treatments, including ovulation induction (OI) and assisted reproductive techniques (ART), could be associated with an increased risk of benign breast diseases (BBDs). To date, no studies have been conducted on this association. Therefore, the present study investigated the association between different types of infertility treatments and BBDs.

**Methods:**

This case‒control study was conducted in Arash Women’s Hospital, Tehran, Iran. The case group included infertile women diagnosed with BBDs without atypia, and the control group included infertile women without breast disease. Breast imaging studies (mammography/ultrasound) were performed for BBD screening, and the diagnosis was confirmed by histopathological examination. Study variables were collected retrospectively from medical records, hospital databases, and questionnaires.

**Results:**

Finally, 154 infertile women, including 50 cases (BBDs) and 104 controls (no BBDs), were compared. Our data showed that 66% of cases and 61.4% of controls had undergone at least one course of infertility treatment. There was no association between BBD risk and previous infertility treatments (OR = 1.21; 95% CI = 0.59–2.46), ART (OR = 1.14; 95% CI = 0.90–1.44), or OI cycles (OR = 1.13; 95% CI = 0.98–1.32). Stratification by confounding variables did not change these results.

**Conclusions:**

It seems that there is no association between BBDs in infertile women and the type, duration, or number of prior infertility treatments; however, considering the small sample size of the study, the clinical significance of this finding should not be neglected. Therefore, we consider it essential to carry out more extensive, detailed, and prospective studies to distinguish the association of BBDs with different infertility treatments and medications.

## Introduction

Benign breast diseases (BBDs) are nonmalignant diseases of the breast that can affect women of all ages, from early reproductive to postmenopausal [1]. The incidence of BBDs is approximately ten times that of breast cancer, yet they have been studied much less frequently. Among BBDs, fibroadenoma is the most common, occurring in 25% of women [2]. Proliferative forms of BBDs have been shown to be associated with an increased risk of developing breast cancer, particularly when atypia is present [3, 4]. Identifying risk factors for BBDs and implementing appropriate management strategies for these benign conditions will ultimately reduce the risk of premalignant and malignant disease. Additionally, the diagnosis of a breast lesion, even if benign, often requires invasive diagnostic procedures such as needle or vacuum-assisted biopsy, which must be followed up regularly and represent a significant psychological burden and anxiety for affected women [5, 6].

Despite increasing evidence supporting the association of some BBDs with breast cancer risk and the rising incidence of BBDs [7, 8], there is still insufficient evidence on the etiology and risk factors for BBDs, as most studies in the field of breast disease are mainly focused on breast malignancies. The breast, being a hormone-sensitive tissue, can be affected by hormonal fluctuations, disrupting its normal function and growth. Various reproductive and hormonal factors, such as parity, age at first pregnancy, estrogen dominance (a condition where estrogen levels are relatively high compared to progesterone), obesity, hormone replacement therapy, polycystic ovary syndrome (PCOS), early menarche, and late menopause, can influence breast health [9, 10]. Given the nature of infertility and the endocrinologic environment associated with its treatment, infertile women may theoretically be at higher risk for breast pathologies.

While this study focuses on the association between infertility treatments and BBDs, it is pertinent to acknowledge the existing literature on malignant breast diseases. Several studies have explored the relationship between infertility treatments and the risk of breast cancer, with varying conclusions. Some have suggested a potential increase in risk, particularly with certain forms of assisted reproductive technology (ART), while others have found no significant association [11–16]. These mixed findings highlight the complexity of the relationship between hormonal interventions and breast tissue pathology. Understanding the exact relationship between hormonal fluctuations caused by infertility or its treatment and BBDs is even more complicated, due to the limited study of BBD’s etiology and risk factors and the heterogeneous nature and the classification of BBDs.

According to our literature review, there are no studies on the association between infertility treatments and the risk of BBDs. This study aims to fill this gap by specifically examining the link between infertility treatments and BBDs, which, despite being more prevalent, have received less attention in the scientific literature. Infertility treatments can vary in their invasiveness and dosage of drugs. Ovulation induction (OI) aims at monofollicular induction and involves lower doses of drugs and the least invasive treatment options, while the aim of ART and controlled ovarian stimulation (COS) cycles is to induce superovulation (a process where hormonal medications are used for stimulation of multiple follicles to develop simultaneously) or and requires different injectable medications at higher doses [17]. So, this study aims to assess the association of BBDs with previous infertility treatments, including OI, ART, and exogenous hormonal exposures separately. We hope to fill a gap in the understanding of how reproductive interventions may influence breast health in a broader sense.

## Materials and methods

### Design and settings

This case‒control study was conducted in the infertility clinic and breast clinic of Arash Women’s Hospital, Tehran University of Medical Sciences, Tehran, Iran, from April 2021 to May 2022.

### Ethics

The present study was designed and conducted in accordance with the Declaration of Helsinki, and the study protocol was approved by the research ethics committees of Tehran University of Medical Sciences, Tehran, Iran (ethics approval code: IR.TUMS.MEDICINE.REC.1399.1072). Written informed consent was obtained from all participants before the study.

#### Confidentiality and data privacy

Participant confidentiality and data privacy were ensured throughout the study. Written informed consent was obtained, and all data were anonymized and securely stored. Data were only accessible to the research team, and all electronic records were password-protected. Physical records were stored in a locked cabinet in a secure location. Participants were assigned unique identification codes to maintain anonymity, and all identifying information was removed from the dataset before analysis.

### Participants

**Fig. 1:**
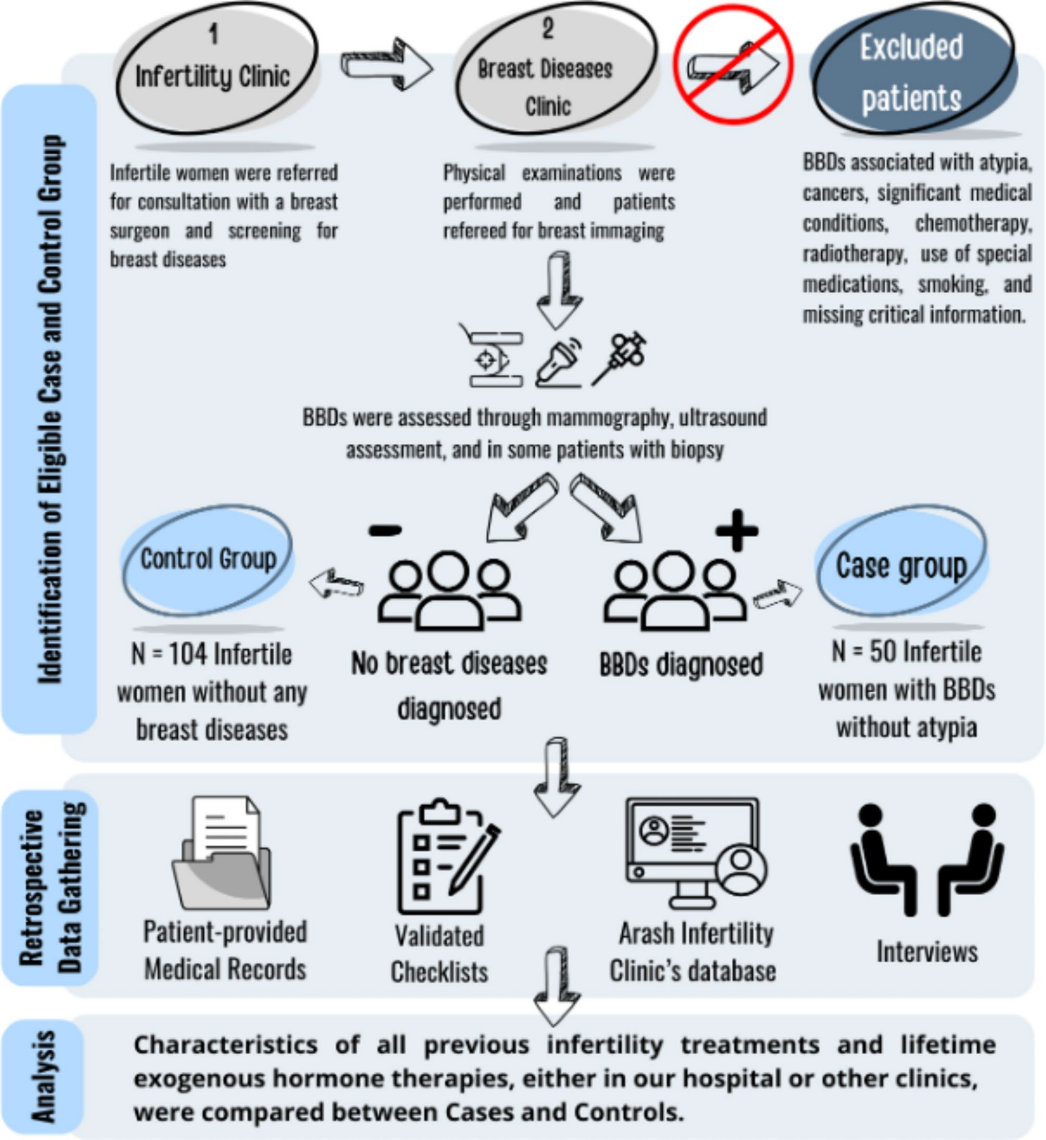
Schematic flow of the study

The study flow diagram is shown in Fig. 1. A total of 154 infertile women were selected from infertile women referred to the Breast Clinic of Arash Women’s Hospital for screening of breast diseases. Infertility was defined as the failure to establish a clinical pregnancy after 12 months of regular, unprotected sexual intercourse.

#### Inclusion criteria

Infertile women aged 18–42 years.

BMI between 18.5 and 29.9.

Regular menstrual cycles.

No previous history of breast diseases.

#### Exclusion criteria

BBDs associated with atypia.

Previous or recent cancer (including breast cancer and other cancers).

History of hormone replacement therapy.

Cigarette smoking.

Presence of other significant medical conditions (e.g., uncontrolled diabetes, severe cardiovascular diseases).

Use of medications that could affect breast tissue, excluding sex hormones and infertility treatments (e.g., long-term corticosteroids, certain antipsychotics, and immunosuppressants).

Missing critical information necessary for the analysis, such as key medical and reproductive history or comprehensive treatment records.

### Recruitment

The case group (*n* = 50) consisted of infertile women who were diagnosed with BBD as defined below, and the control group (*n* = 104) consisted of infertile women with no breast lesions, confirmed by the same diagnostic procedures used for the case group.

#### Diagnosis of BBDs

Breast disease was evaluated according to the usual clinic program by physical examination and ultrasound in all women and additionally by mammography in women older than 40 years. When necessary, a core needle biopsy was performed by the breast surgeon for palpable lesions and under ultrasound guidance for nonpalpable findings. The diagnosis of BBD was confirmed by the breast surgeon after complete examination and included breast cysts greater than 20 mm in diameter detected on ultrasound or physical examination and confirmed histologically or ductal hyperplasia detected on histological examination.

**Criteria for defining type of BBDs**

- Breast Cysts: Cysts greater than 20 mm in diameter detected on ultrasound or physical examination and confirmed histologically.
- Ductal Hyperplasia: Detected on histological examination.
- Fibroadenoma: Typically identified as a firm, rubbery, and movable lump during physical examination. Confirmed by ultrasound and histological examination following a biopsy.
- Duct Ectasia: Diagnosed through clinical symptoms such as nipple discharge and pain, supported by imaging findings on mammography or ultrasound, and confirmed by histological examination.
- Intraductal Hyperplasia: Diagnosed through histological examination of breast tissue samples obtained via biopsy. Usual ductal hyperplasia (UDH) shows an overgrowth of normal-looking cells lining the ducts, while atypical ductal hyperplasia (ADH) shows more distorted and abnormal cells.

### Data collection

This study gathered information about a range of medical and laboratory interventions aimed at treating various forms of female infertility. These include all types of ovulation induction (OI), intrauterine insemination (IUI), and assisted reproductive technology (ART) (including controlled ovarian stimulation [COS], in vitro fertilization [IVF], intracytoplasmic sperm injection [ICSI], and frozen-thawed embryo transfer [FET]).

#### Data collection methods

1. *Medical Records*: Data were retrospectively gathered from the Arash Infertility Clinic’s database and patient-provided records. Standardized data extraction forms were used to ensure consistency across all records.
2. *Checklists*: Participants provided information through a custom-designed checklist that captured various demographics and medical information. This checklist was completed either during their clinic visit or at home, providing flexibility for participants.
3. *Interviews*: Structured interviews were conducted by trained research assistants who followed a standardized interview protocol to ensure consistency. These interviews were designed to gather detailed information on participants’ medical history, treatment experiences, and outcomes.

#### Data collection standardization

##### Training

All research assistants and personnel involved in data collection underwent rigorous training to ensure they adhered to the standardized protocols.

##### Validation

Although the checklist was custom-designed, it was carefully reviewed and tested to ensure it captured all necessary information accurately. The validity of the checklist was assessed and confirmed through reviews by experienced specialists in infertility, breast diseases, surgeons, pathologists, radiologists, gynecologists, and epidemiologists.

#### Data scope

Our data collection was comprehensive, encompassing all forms of lifetime exogenous hormone therapy. This included oral contraceptive pills (OCP), estradiol, and progesterone, which were prescribed for a variety of clinical indications. These ranged from infertility treatment programs to endometrial preparation for frozen embryo transfer, cycle scheduling, and treatment of gynecological pathologies.

### Infertility treatment protocols

While our hospital’s protocols are well-documented, some participants may have undergone OI or ART at other centers, with incomplete records on our end. Therefore, we’ve collected detailed information about the ART and OI cycles conducted at our hospital but we reported previous infertility treatments history and hormone therapies either in our hospital or in other centers collectively.

The inclusion of various infertility treatments such as OI, COS, and ART is crucial to understand their potential impact on the risk of BBDs. These treatments involve different hormonal regimens and dosages, which may influence breast tissue differently. By examining these treatments, we aim to identify specific infertility protocols that may be associated with an increased risk of BBDs, thereby contributing to better management and prevention strategies for women undergoing fertility treatments.

Then, based on the medical treatment type, participants with history of infertility treatments, were categorized into two subgroups: (1) OI subgroup, either followed by a natural insemination or IUI, and (2) ART subgroup, including COS (either inseminated by ICSU or IVF) and FET)

#### OI subgroup

In OI cycles, treatment commenced with Clomiphene Citrate at a daily dose of 50–100 mg or Letrozole at a daily dose of 2.5-5 mg for 5 consecutive days starting from the 2nd or the 3rd day of the menstrual cycle. Subsequent ultrasound monitoring was performed, and based on the patient’s baseline characteristics (age, ovarian reserve, gynecologic conditions like PCOS, etc.) and ovarian response, the cycle was either continued with Clomiphene Citrate or Letrozole, or a single injection of gonadotropin at a dose of 75 IU might have been administered to further stimulate the development of ovarian follicles. Alternatively, in case of adequate ovarian response, the cycle was followed up without any further intervention until the detection of the dominant follicle. Once the dominant follicle reached a size of ≥ 18 mm, as detected via ultrasound, a subsequent injection of 5,000 to 10,000 IU HCG was administered. Following the HCG injection, patients could opt for spontaneous insemination, which relies on natural conception, or proceed with IUI (direct insertion of washed sperm into the uterus).

It is important to note that IUI cases were only considered eligible if they received OI treatment, thereby maintaining the focus of the study on the potential effects of hormonal changes induced by OI on the risk of BBDs.

#### ART subgroup

**COS cycles**

Patients underwent a Gonadotropin-Releasing Hormone (GnRH) antagonist regimen. This involved 21 days of OCPs pre-treatment in some cases, followed by a series of Gonadotropin injections, either Follicle-Stimulating Hormone (FSH) or Human Menopausal Gonadotropin (HMG), administered at a dosage of 150–300 IU/day for 5 days starting from the third day of the cycle. These hormones could be administered either as individual agents or in combination. The dosage was customized during follow-up visits to align with the patient’s ovarian response. A GnRH antagonist (usually 0.25 mg/day Cetrotide) was introduced when at least two follicles reached 14–15 mm. Oocyte retrieval occurred 36 h after administering 10,000 IU Human Chorionic Gonadotropin (HCG) once follicles were ≥ 18 mm. In cases with a risk of ovarian hyperstimulation syndrome (OHSS), final follicle maturation was triggered with GnRH agonist injection. After oocyte retrieval, the oocytes were fertilized either through conventional IVF or by ICSI.

**FET cycle**

Participants received hormone replacement therapy before FET. They took 6 mg/day of oral *Estradiol Valerate* (EV) (2 mg film-coated tablets, *Abureyhan* Pharmaceutical Co., Iran) from cycle day 2 to promote endometrial growth. Endometrial thickness was monitored via ultrasound, and EV dosage was adjusted accordingly. Luteal phase support was initiated once the endometrium reached favorable thickness ( > = 7 mm) and pattern by taking 800 mg/day *progesterone* suppository (*cyclogest* 400 mg vaginal suppositories, *Actoverco* Pharmaceutical Co., Iran) or receiving 50 mg/day intramuscular (IM) progesterone injections (*Fertigest*, *Aburaihan* Pharmaceutical Co., Iran). Luteal phase support was followed by after embryo transfer until pregnancy confirmation.

### Study variables

For data presentation and analysis, study variables were divided into baseline variables (including demographic characteristics [age and body mass index], reproductive history [obstetric, infertility, and menstrual history], and family history), cause of infertility, and characteristics of previous infertility treatments and exogen hormone therapies. In this study, we categorized infertility causes into three primary groups: Anovulation/hormonal causes, non-hormonal causes, and non-female causes. These categories were defined as follows:

Anovulation/hormonal causes stands for the four most common ovulatory disorders, including polycystic ovary syndrome, hypogonadotropic hypogonadism, primary ovarian insufficiency, and hyperprolactinemia; nonhormonal causes include endometriosis, tubal factor anatomical abnormalities and other female causes of infertility. Non-female cause included male factor (infertility related to male reproductive issues, including but not limited to low sperm count, poor sperm motility, abnormal sperm morphology, and obstructive problems) or unexplained infertility (cases where comprehensive testing has not identified a clear cause of infertility in either partner).

### Handling missing data

Missing data were addressed by excluding cases with incomplete records from external centers. Specifically, cases were excluded if they lacked critical information necessary for the analysis, such as key medical and reproductive history, or comprehensive treatment records. However, if only specific details such as the brands or precise dosages of hormonal agents used in external centers were not available, these cases were not excluded. No imputation methods were used.

### Statistical analysis

All analyses were performed with SPSS Statistics for Windows, version 23.0 (IBM Corp., Armonk, NY, USA). All p values were considered significant at *p* < 0.05.

#### Continuous variables

Presented as the means ± standard deviations (SD) and were compared using Student’s t test. The choice of the student’s t-test was based on the assumption of normal distribution for continuous variables.

#### Categorical variables

Presented as frequencies (percentages), and their differences were tested with the chi-square test or Fisher’s exact test for small numbers, whichever was appropriate.

#### Logistic regression

Logistic regression analysis was used to estimate the odds ratios (ORs) for the association between infertility treatments and breast benign disorders, adjusting for potentially confounding effects of age, gravidity, parity, age at first pregnancy, and age at first birth. Given the case-control design of our study, logistic regression was chosen to model the relationship between exposure (infertility treatments) and outcome (breast benign disorders) while controlling for confounders. This approach allows for the estimation of adjusted odds ratios, providing a clearer understanding of the associations.

Confounding variables were selected based on their potential impact on the risk of breast benign disorders (BBD) and their ability to confound the relationship between infertility treatments and BBD. The selection criteria included:

1. *Prior studies*: Variables such as age, gravidity, and parity have been identified in the literature as important factors influencing the risk of breast diseases. We reviewed relevant studies to identify commonly controlled confounders in similar research.
2. *Theoretical considerations*: The selection was guided by theoretical frameworks that highlight the potential impact of these variables on breast health. For example, age is a well-known factor influencing breast tissue changes, while gravidity and parity provide insights into hormonal influences on breast tissue.

## Results

### Baseline characteristics

**Fig. 2:**
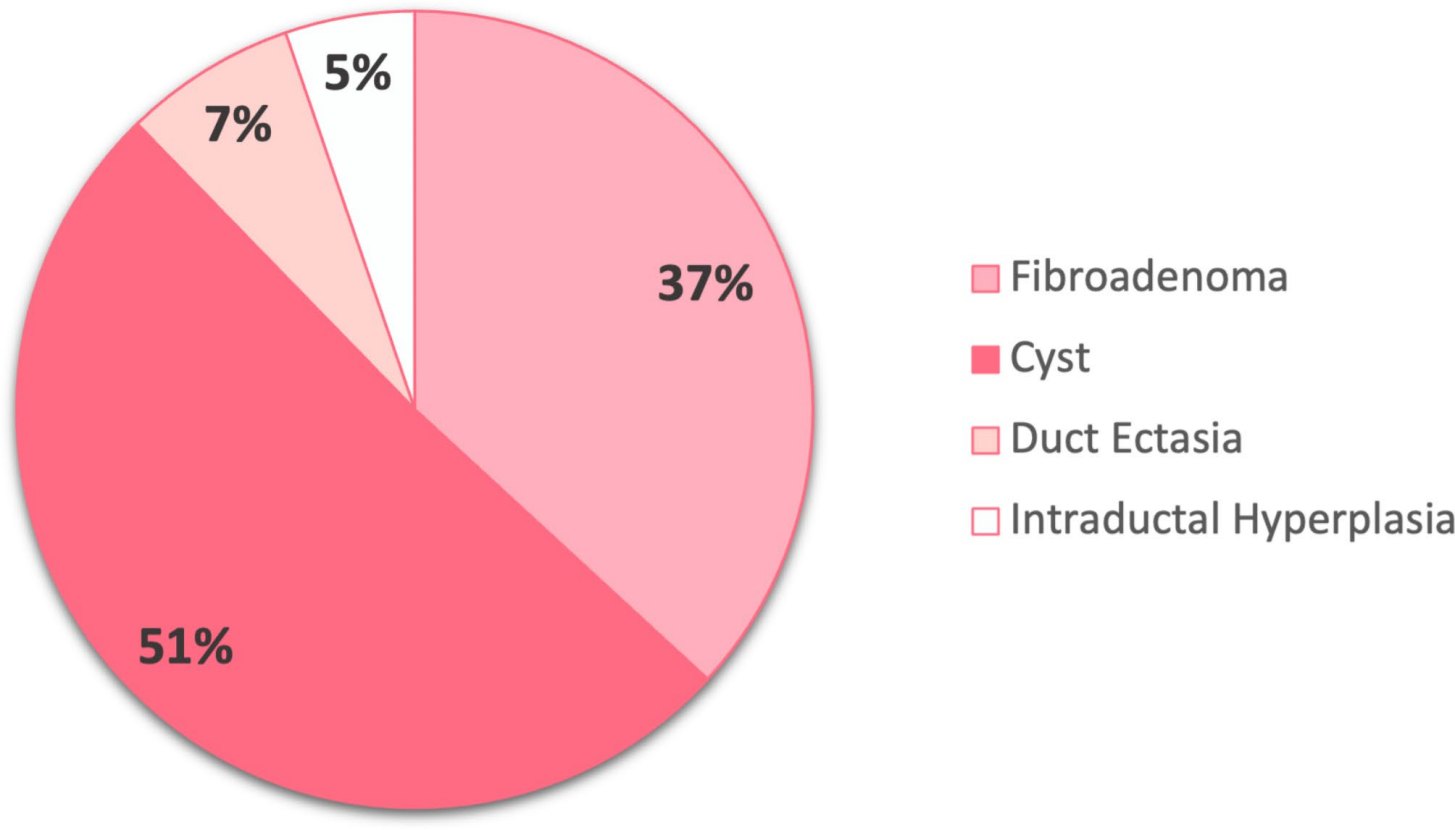
Overall frequency of different benign lesions

The association between BBDs and previous infertility treatments was evaluated among a total of 154 infertile women, including 50 cases diagnosed with BBD and 104 controls without BBDs. Figure 2 illustrates the prevalence of the different types of BBDs among the participants.

The participants’ baseline characteristics are shown in Table 1. The mean age of women with BBD was significantly higher than that of the control group (33.88 ± 4.04 years vs. 31.73 ± 4.57 years, *P* = 0.005). This suggests that age may be a factor in the development of BBDs. A higher proportion of women with BBD had been pregnant at least once compared to those without BBD (55% vs. 40%, *P* = 0.053), indicating a potential link between pregnancy history and BBDs. Women with BBD were less likely to report irregular menstruation compared to those without BBD (14% vs. 35.6%, *P* = 0.006). Other baseline characteristics did not show significant differences between the groups.

**Table 1:** Baseline characteristics

		No BBD (*n* = 104)	BBD (*n* = 50)	*P* value
**Demographic Characteristics**				
Age (years) ^a, b^		31.73 ± 4.57	33.88 ± 4.04	**0.005**
	< 25	14 (13.5)	2 (4)	0.067
	25–30	22 (21)	9 (18)	
	30–35	45 (43.5)	19 (38)	
	> 35	23 (22)	20 (40)	
BMI (kg/m^2^) ^a^		25.99 ± 4.51	25.85 ± 3.82	0.847
**Reproductive History**				
Gravidity ^b^				
	0	63 (60)	22 (45)	0.053
	>=1	41 (40)	28 (55)	
Age at first pregnancy ^b^				
	< 25	19 (47.5)	10 (36)	0.368
	25–30	17 (40)	11 (39)	
	> 30	5 (12.5)	7 (25)	
Parity ^b^				
	0	83 (80)	33 (65)	0.063
	>=1	21 (20)	17 (35)	
Age at first parturition ^b^				
	< 25	12 (57)	6 (35)	0.369
	25–30	8 (38)	9 (53)	
	> 30	1 (5)	2 (12)	
Miscarriage history ^c^		29 (28)	16 (32)	0.365
Breastfeeding history ^b^				
	Never	85 (81.5)	34 (68)	0.280
	< 6 Months	3 (3)	2 (4)	
	6–12 Months	0	0	
	13–24 Months	13 (12.5)	12 (24)	
	> 25 Months	3 (3)	2 (4)	
Infertility Duration (years) ^a^		6.04 ± 4.00	5.73 ± 3.67	0.650
Infertility Type ^c^				
	Primary	63 (60.5)	22 (45)	0.053
	Secondary	41 (39.5)	28 (55)	
Cause of Infertility ^b, *^				
	Anovulation/hormonal causes	44 (43)	14 (28)	0.329
	Nonhormonal causes	21 (20)	13 (26)	
	Nonfemale causes	39 (37.5)	23 (46)	
Age at Menarche ^a^		12.92 ± 1.95	12.96 ± 1.61	0.908
Irregular Menstruation ^b^		37 (35.6)	7 (14.0)	**0.006**
Menstrual abnormalities				
	Amenorrhea/Oligomenorrhea ^b^	16 (15.4)	5 (10)	0.362
	Menorrhagia/Metrorrhagia ^b^	14 (13.5)	4 (8)	0.323
**Family History**				
BC History in First Relatives ^b^		4 (3.8)	2 (4)	0.963
OC History in First Relatives ^b^		1 (1)	0	0.487

a: data presented as the mean ± standard deviation and analysis was based on T test; b: data presented as number (%) and analysis was based on Chi-squared or c: Fisher’s exact test; * Anovulation/hormonal causes stands for the four most common ovulatory disorders, including polycystic ovary syndrome, hypogonadotropic hypogonadism, primary ovarian insufficiency, and hyperprolactinemia; nonhormonal causes include endometriosis, tubal factor anatomical abnormalities and other female causes of infertility. Non-female cause included male factor or unexplained infertility. BMI: body mass index; BC: breast cancer; OC: ovarian cancer

### Infertility treatment history

**Fig. 3:**
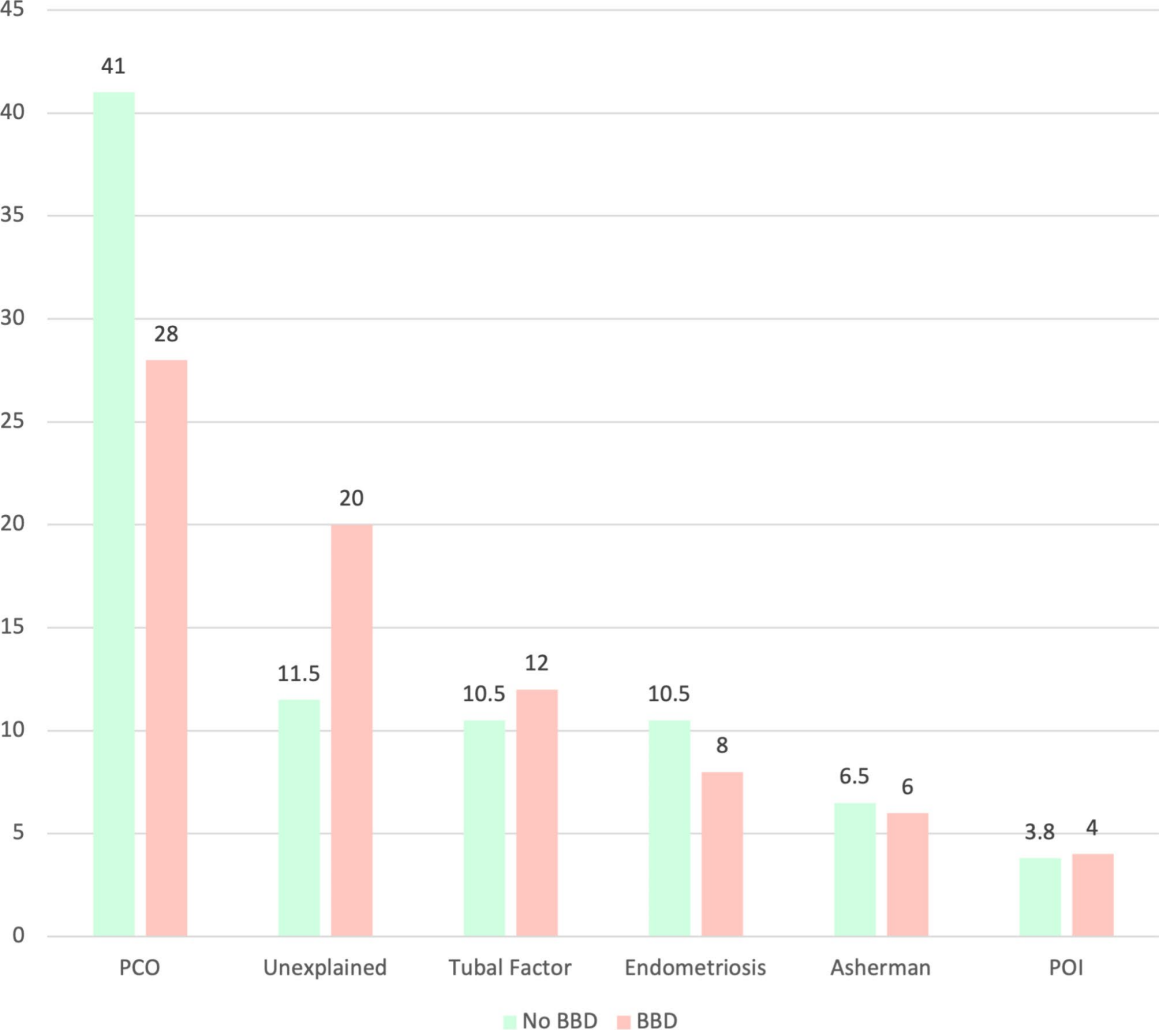
Detailed causes of female factor infertility. PCOS: Polycystic Ovary Syndrome; POI: Primary ovarian insufficiency

Details of the causes of infertility are shown in Fig. 3 and compared between groups. In Table 2, the characteristics of previous infertility treatments, including their number, type, and lifetime hormone therapy history, were compared between the two groups, and no significant differences were found. An equal number of participants in the control and case groups had a history of infertility treatments, either by OI or ART (61.5% vs. 66%).

**Table 2:** Characteristics of previous infertility treatments

		No BBD (*n* = 104)	BBD (*n* = 50)	*P*-value
**Total Infertility Treatments**				
History of previous infertility treatments ^a^		64 (61.5)	33 (66)	0.591
Total number of infertility treatments cycles ^b^		2.59 ± 2.39	3.52 ± 3.79	0.120
infertility treatments type ^a^				
	ART ^#^	52 (81)	25 (76)	0.526
	OI ^‡^	12 (19)	8 (24)	
**ART Treatments Characteristics**				
Total Number of ART cycles ^b^		1.17 ± 1.30	1.44 ± 1.64	0.317
Last ART cycle ^a^				
	=< 1 year	24 (46)	12 (48)	0.809
	1–2 years	10 (19)	6 (24)	
	>= 3 years	18 (35)	7 (28)	
**Lifetime Exogenous Hormone Therapy** ^**†**^				
Total courses of OI ^b^		1.42 ± 1.72	2.08 ± 3.01	0.157
Total courses of hMG or FSH injection ^*^		1.62 ± 1.05	1.56 ± 0.97	0.773
Progesterone use history ^b^		61 (58.7)	33 (66)	0.381
Progesterone use duration (months) ^a^		1.55 ± 2.56	1.95 ± 2.62	0.379
EV use history ^b^		59 (56.7)	27 (54)	0.749
EV use duration (months) ^a^		1.70 ± 4.07	1.72 ± 3.38	0.984
OCP use History ^b^		54 (52)	26 (52)	0.993
OCP use duration (months) ^a^		7.00 ± 17.84	7.88 ± 18.53	0.780

a: Data presented as ratio (%) and analysis were based on chi-squared; b: Data presented as the mean ± standard deviation and analysis were based on T test; # Including COS cycles (either inseminated by ICSI or IVF) and FET; ‡ Including OI by Clomiphene citrate or Letrozole that were followed either by natural insemination or combined with IUI; † This data is related to all forms of lifetime exogenous hormone therapy. This included OCP, EV, and progesterone, which were prescribed for a variety of clinical indications. These ranged from infertility treatment programs to endometrial preparation for frozen embryo transfer, cycle scheduling, and treatment of gynecological pathologies. * Includes history of all COS cycles that required courses of hMG or FSH injection. ART: Assisted reproductive techniques; CI: Confidence interval; COS: controlled ovarian stimulation; EV: estradiol Valerate; FET: Frozen-thawed embryo transfer; FSH: Follicle Stimulating Hormone; hMG: human Menopausal Gonadotropin; ICSI: Intracytoplasmic injection; IUI: intrauterine insemination; IVF: In vitro fertilization; OCP: oral contraceptive pills; OI: Ovulation induction; OR: odds ratio

### Univariate analysis results

The results of the univariate analysis are shown in Table 3 and revealed no association between previous infertility treatments and the risk of BBDs. Stratifying by gravidity, parity, age at first pregnancy, and age at first parturition did not substantially affect these results.

**Table 3:** Association of fertility treatments with the risk of BBD in infertile women

	Univariate OR (95% CI)	*P* value	Multivariate OR (95% CI) ^a^	*P* value
History of previous infertility treatments	1.21 (0.59–2.46)	0.592	4.06 (0.74–22.03)	0.104
Total number of infertility treatments cycles	1.10 (0.98–1.24)	0.077	1.17 (0.83–1.63)	0.355
Number of ART cycles ^#^	1.14 (0.90–1.44)	0.276	1.37 (0.76–2.46)	0.292
Number of OI cycles ^‡^	1.13 (0.98–1.32)	0.099	1.09 (0.71–1.68)	0.684

a: ORs and 95% CI adjusted for age, G, P, age at first pregnancy, and age at first parturition; # Including COS (either inseminated by ICSI or IVF) and FET; ‡ including OI cycles, either followed by natural insemination or combined with IUI; ART: Assisted reproductive techniques; OI: Ovulation induction; ICSI: Intracytoplasmic injection; IVF: In vitro fertilization; FET: Frozen-thawed embryo transfer; IUI: intrauterine insemination; OR: odds ratio; CI: Confidence interval

## Discussion

The present study demonstrated that there is no significant association between BBDs and the type, duration, or number of previous infertility treatments. One interesting finding of our study was the observation of a higher number of total OI cycles in the case group (2.08 ± 3.01) compared to the control group (1.42 ± 1.72), although this difference did not reach statistical significance (*P* = 0.157). Despite the lack of statistical significance, this result may still hold clinical relevance. The higher number of OI cycles could suggest a trend that, with a larger sample size, might reveal a significant association. This trend is worth considering because it may indicate underlying biological or clinical factors that contribute to the development of BBDs in women undergoing infertility treatments.

For instance, repeated OI cycles could lead to prolonged exposure to elevated levels of estrogen and progesterone, which are known to influence breast tissue proliferation. Even if the current study’s sample size was insufficient to detect a statistically significant difference, the observed trend aligns with the hypothesis that hormonal fluctuations associated with infertility treatments could impact breast health. Therefore, these findings highlight the need for further research with larger sample sizes to explore this potential association more thoroughly.

Comprehensive searches revealed that epidemiologic studies mainly focused on the association between infertility and malignancies rather than BBDs. We did not find any studies that examined the association between infertility treatments and BBDs, so we could not directly compare our results with other studies. Therefore, we discuss the results of this study mainly on the basis of studies on the association between hormonal factors and the risk of BBDs.

It has been proposed that changes in the ratio of estradiol and progesterone that occur during ovulatory cycles are responsible for the development and relapse of BBDs. Breast benign proliferative disorders are more common in women with higher serum estradiol levels, but other biochemical disturbances may also play a role. For example, higher serum levels of insulin, CRP, and adiponectin are other independent risk factors for benign breast proliferative lesions. Therefore, complicated endocrinological and inflammatory pathways are involved in breast tissue proliferation [18].

Almost all medications used in infertility treatments could result in a temporary but significant elevation of circulating estrogen and progesterone. Therefore, hyperestrogenemia caused by superovulation and administration of exogenous estradiol for endometrial preparation during frozen embryo transfer or for other purposes could theoretically influence the risk of BBDs. On the other hand, the transient nature of hormonal fluctuations induced by infertility treatments might not be sufficient to cause long-term changes in breast tissue. While these treatments involve significant hormonal manipulation, the duration and intensity of exposure may not reach the threshold required to influence the development of BBDs.

Hormonal risk factors for BBDs have been shown to vary depending on the histopathologic subtype of the lesions. For example, current or previous use of OCPs has been shown to be significantly associated with an increased risk of fibroadenoma and a decreased risk of breast epithelial proliferation without atypia but not with a risk of breast cysts or fibrocystic changes [19]. In this study, we found no differences between cases and controls with respect to history of OCP, estradiol, or progesterone use.

It has been proposed that the association of breast disease (either malignant or benign types) and infertility may be due to infertility itself rather than the drugs used to treat it. There are studies supporting the role of infertility in providing a background for further complications and mortalities in affected women, as they are at increased risk of breast cancer and cancer-related death [15, 20, 21]. In contrast, some other studies showed that there is no association between infertility and breast cancer risk [22].

In addition to infertility treatments, infertile women have other risk factors for breast disease, such as reproductive history (nulliparity, older age at first pregnancy, not breastfeeding) or endogenous/exogenous hormonal stresses (menstrual irregularities, anovulation, PCOS). Recently, a large cohort study of 61,617 women found that age and family history of breast cancer, known to be associated with breast cancer risk, were also associated with the risk of BBDs [19]. This finding was consistent with the results of the present study, as the age of women with BBDs was significantly higher than that of the control group. However, after controlling for gravidity, parity, and age at first pregnancy and delivery, this finding was not statistically significant.

One of the interesting findings of the present study was the lower rate of nulliparity and nulligravidity in infertile women diagnosed with BBDs. These findings were not statistically significant; however, they were opposed to the results of older studies indicating a protective effect of pregnancy on the risk of breast cancer [23] and BBDs [24, 25].^19^ Several factors could account for these unexpected results. Firstly, the small sample size of our study may limit the statistical power to detect significant differences and could contribute to these discrepancies. Secondly, population differences, such as age, genetic, hormonal, environmental, or lifestyle factors, might influence the prevalence and risk factors for BBDs in our study cohort compared to those in previous studies. Lastly, methodological considerations, including differences in study design, data collection, and analysis techniques, could also play a role in these contrasting findings.

Another important finding of our study was the lower prevalence of menstrual disorders in the case group. Johansson et al. [19] also concluded that regular menstrual cycles were associated with an increased risk of fibroadenomas in the premenopausal age group but not with other types of BBDs. This finding may be related to the fact that higher in situ concentrations of estrone and estradiol [26] and higher expression of estradiol and progesterone receptors [27] are observed in fibroadenoma tissue compared with the rest of the breast tissue. However, similar to the previous point, differences in sample size, population characteristics, and methodological approaches could explain the discrepancies between our findings and existing literature.

### Clinical and public health implications

Our findings suggest that while there is no significant association between infertility treatments and BBDs, the observed trends highlight the need for careful monitoring of women undergoing these treatments. Clinicians should be aware of the potential for increased OI cycles to influence breast health and consider this when counseling patients about the risks and benefits of infertility treatments. Additionally, these results underscore the importance of personalized treatment plans that take into account the patient’s reproductive history and hormonal exposure.

### Recommendations for future research

Future studies should focus on prospective cohort designs with larger sample sizes to validate these findings and explore the potential associations more thoroughly. Including specific variables such as detailed hormonal profiles, genetic predispositions, and lifestyle factors could help clarify the relationship between infertility treatments and BBDs. Additionally, investigating the long-term effects of different infertility treatment protocols on breast health will be crucial in developing guidelines for safer reproductive health practices.

### Study limitations and strengths

The retrospective nature of this study may introduce inherent constraints, particularly in controlling for the specific medications utilized by participants. This variability reflects the individualized approach to infertility treatment and the diversity of clinical practices among different centers. Due to the retrospective design, comprehensive details such as the brands or precise dosages of hormonal agents were not consistently available for all participants, leading to recall bias.

The heterogeneity of interventions, a common bias in surveys assessing the risks of infertility treatments, was also present in our study. Our treatment protocols necessitated varying combinations of drugs, dosages, and durations to optimize ovarian response.

Furthermore, our primary objective was to explore the extended impact of lifetime infertility treatments on breast disease development. Acknowledging that breast disease manifestations may be delayed, we considered the full spectrum of hormonal and infertility treatments received by participants, extending beyond the treatments administered at our facility. This comprehensive approach is vital for understanding the risk factors linked to benign breast diseases.

The small sample size is another limitation which may result in over- or underestimation of the true associations between infertility treatments and breast disease. This limitation also can affect the generalizability of our findings, as the study population may not be representative of the broader population undergoing infertility treatments. Consequently, our conclusions should be interpreted with caution, and further research with larger, more diverse cohorts is necessary to validate our findings.

A key strength of our study is the consistent application of treatment protocols for participants undergoing OI or ART at our center. However, we also included data from clinics outside of our center, which introduces variability in treatment protocols. Generally, uniform treatment approaches, like the ones used at our center, have both benefits and limitations. On the positive side, they ensure consistency and reliability in the findings, as all participants receive the same standardized care. This can reduce variability and make it easier to interpret the results. However, the downside is that such uniformity might limit the applicability of the findings to other settings where different protocols are used. This dual nature of standardized protocols should be considered when interpreting the results and their potential impact on clinical practice.

Despite all mentioned limitations, our study provides a valuable foundation for future research. It highlights the need for further investigation into the relationship between infertility treatments and breast health, and identifies gaps in current knowledge that future studies can address.

## Conclusion

This study found no statistically significant association between BBDs and medical infertility treatments. However, due to the study’s heterogeneity and small sample size, these results should be interpreted with caution. The observation of a nonsignificant higher number of total OI cycles in cases with BBDs suggests potential clinical importance, warranting further investigation.

Future research should focus on larger, more detailed, and prospective studies to better understand the association between BBDs and infertility treatments. These studies should aim to distinguish the effects of infertility itself from those of the treatments, considering the limitations identified in this study.

## Data Availability

The datasets used and/or analysed during the current study are available from the corresponding author on reasonable request.
